# Combining Activity Trackers With Motivational Interviewing and Mutual Support to Increase Physical Activity in Parent-Adolescent Dyads: Longitudinal Observational Feasibility Study

**DOI:** 10.2196/pediatrics.8878

**Published:** 2018-04-12

**Authors:** Josette Bianchi-Hayes, Elinor Schoenfeld, Rosa Cataldo, Wei Hou, Catherine Messina, Susmita Pati

**Affiliations:** ^1^ Department of Pediatrics Stony Brook University & Stony Brook Children's Hospital Stony Brook, NY United States; ^2^ Department of Family, Population, & Preventive Medicine Stony Brook University Stony Brook, NY United States

**Keywords:** adolescent obesity, activity trackers, dyads, motivation, physical activity, adolescent health, pediatric obesity, fitness trackers, parent-child relations, motivation, exercise

## Abstract

**Background:**

An essential component of any effective adolescent weight management program is physical activity (PA). PA levels drop dramatically in adolescence, contributing to the rising prevalence of adolescent obesity. Therefore, finding innovative interventions to address this decline in PA may help adolescents struggling with weight issues. The growing field of health technology provides potential solutions for addressing chronic health issues and lifestyle change, such as adolescent obesity. Activity trackers, used in conjunction with smartphone apps, can engage, motivate, and foster support among users while simultaneously providing feedback on their PA progress.

**Objective:**

The objective of our study was to evaluate the effect of a 10-week pilot study using smartphone-enabled activity tracker data to tailor motivation and goal setting on PA for overweight and obese adolescents and their parents.

**Methods:**

We queried enrolled adolescents, aged 14 to 16 years, with a body mass index at or above the 85th percentile, and 1 of their parents as to behaviors, barriers to change, and perceptions about exercise and health before and after the intervention. We captured daily step count and active minutes via activity trackers. Staff made phone calls to dyads at weeks 1, 2, 4, and 8 after enrollment to set daily personalized step-count and minutes goals based on their prior data and age-specific US national guidelines. We evaluated dyad correlations using nonparametric Spearman rank order correlations.

**Results:**

We enrolled 9 parent-adolescent dyads. Mean adolescent age was 15 (SD 0.9) years (range 14-16 years; 4 female and 5 male participants); mean parent age was 47 (SD 8.0) years (range 36-66 years). On average, adolescents met their personalized daily step-count goals on 35% (range 11%-62%) of the days they wore their trackers; parents did so on 39% (range 3%-68%) of the days they wore their trackers. Adolescents met their active-minutes goals on 55% (range 27%-85%) of the days they wore their trackers; parents did so on 83% (range 52%-97%) of the days. Parent and adolescent success was strongly correlated (step count: *r*=.36, *P*=.001; active minutes: *r*=.30, *P*=.007). Parental age was inversely correlated with step-count success (*r*=–.78, *P*=.01).

**Conclusions:**

Our findings illustrate that parent-adolescent dyads have highly correlated PA success rates. This supports further investigation of family-centered weight management interventions for adolescents, particularly those that involve the parent and the adolescent working together.

## Introduction

Childhood and adolescent obesity have a significant lifelong impact on both the individual and the health care system. The prevalence of adolescent obesity (12-19 years) in the United States is approximately 21%, up from 5% only 30 years ago [1]. Obese adolescents are more likely to have both acute and long-term chronic health problems related to their obesity, including prediabetes, cardiovascular disease, and psychological comorbidities [2].

A crucial component of any effective adolescent weight management program is physical activity (PA) [3]. Yet PA levels drop dramatically in adolescence, contributing to the rising prevalence of adolescent obesity [4-6]. While the US national guidelines typically recommend 60 minutes daily of PA or 12,000 steps per day for children and teens [7-10], research indicates that even a modest amount of PA can have health benefits for high-risk youth [3]. Additionally, studies of step counts have found that a more modest daily step count (10,000-11,700 steps/day) may be appropriate for overweight adolescents [11]. Finding effective and sustainable ways to increase PA among these high-risk youth, at this crucial developmental stage, requires novel and innovative interventions. Nonetheless, despite this growing epidemic, developing effective interventions for this population remains a challenge [12].

The growing field of health technology, including smartphone apps and activity trackers, provides potential solutions for addressing chronic health issues and lifestyle change, such as adolescent obesity. These types of apps appeal to and are accessible to the adolescent demographic. As of 2015, the Pew Research Center found that 73% of US adolescents have access to a smartphone [13]. Additionally, a wealth of options that specifically target increasing PA, such as wearable activity trackers, are gaining popularity [14]. These trackers can be used in conjunction with smartphone apps to engage, foster support, and motivate while simultaneously providing feedback on progress. Many available wearable activity tracker apps include behavioral change techniques such as self-monitoring, feedback, and goal setting but often do not provide sufficient motivation to encourage consistent activity over time [15]. Enhancing these basic behavioral change techniques with personalized, tailored interaction with the study team, as well as participation within a parent-adolescent dyad, is a potential strategy to increase PA in this population.

To effectively leverage this technology, it must be combined with established tenets of childhood obesity management. In addition to the roles of diet and PA, one such tenet is the caregiver’s or parent’s role in weight management [16,17]. In our previous research, we found that caregivers want to be involved in a weight management intervention for their adolescent child, at the level of both development and administration of the weight management intervention [18]. Despite evidence of the important role of the parent in their child’s weight management [16,17], only a small percentage (12.3%) of mobile apps that have addressed pediatric obesity involved the family [19]. To address this growing epidemic of adolescent obesity, we evaluated the feasibility and preliminary efficacy of a novel intervention that combined this critical element of parental support with motivational interviewing techniques driven by participant activity tracker data.

## Methods

### Study Population

Our pilot study recruited parent-adolescent dyads from participants in an earlier caregiver survey study [18] and from 2 ambulatory office sites in our hospital’s catchment area. Eligible adolescents were between 13 and 16 years of age and had a body mass index at or above the 85th percentile for their height and sex. We selected this minimum age because the activity tracker used, Jawbone UP MOVE (Jawbone, San Francisco, CA, USA), is only approved for use by individuals aged 13 years and older. We selected this maximum age because preliminary caregiver survey data [18] suggested that older adolescents’ lifestyles generally would not be conducive to participating in an intervention with their parent. Eligible participants needed to speak English, have a regular health care provider for medical clearance, have access to a smartphone, and be willing to speak to a research assistant on a regular basis.

### Development of Motivational Scripts and Intervention Tools

We used data from an electronic medical record database review, a caregiver survey study, a health care provider focus group, and key informant input from experts in epidemiology, psychology, and pediatric weight management to inform development of the 10-week, multimodal intervention for parent-adolescent dyads. An electronic medical record clinical data analysis of all patients seen within Stony Brook Children’s Hospital’s outpatient pediatric offices (Stony Brook, NY, USA) and inpatient unit in 2013 provided insight into demographics and general trends in the catchment area’s overweight and obese population. Next, a self-administered caregiver survey [18] and a small focus group of pediatric weight management providers helped us better understand the best modes of intervention delivery, potential barriers, and further content development. We reviewed the quantitative and qualitative data from these preliminary steps with key informants to develop the pilot intervention.

We selected the Jawbone UP MOVE activity tracker after carefully reviewing consumer reviews, literature searches [20], and multiple rounds of prepiloting. Additionally, we considered the affordability factor for both the study and accessibility for our patients in the future when selecting the device. According to a study of reliability and validity of several activity trackers, the Jawbone UP was considered reliable (intraclass correlation coefficient between 2 tests was good: 95% CI 0.66-0.91) and valid (achieving a<1% error deviation from the reference standard during walking on a treadmill at a speed of 3 mph) [20]. This study was approved by the Stony Brook University School of Medicine Institutional Review Board prior to enrolling any participants.

### Intervention Design

The 10-week intervention paired overweight and obese adolescents with a parent to participate in daily PA using the Jawbone UP MOVE activity tracker and its associated smartphone app. After a brief eligibility survey administered either by phone or in person by a trained research assistant, parent-adolescent dyads were enrolled in the study. Once they had signed medical clearance forms, enrolled parent-adolescent dyads came to Stony Brook University Medical Center for an in-person orientation with a research assistant. At the orientation, both parents and adolescents were queried about their current PA behaviors, barriers to change, perceptions about exercise and health, sleep patterns, and fitness goals. A similar survey was completed at the end of the 10-week intervention as a debriefing session. We provided both members of the dyad with their own personal activity tracker (Jawbone UP MOVE with wristband) with clear instructions on how to use it. The research assistant configured the activity trackers’ settings and apps to reflect evidence-based, age-appropriate recommendations for goal steps and active minutes [7-10] (see Table 1 for app settings). Privacy and sharing settings and notifications reflected unique barriers and concerns relative to the adolescent population (ie, not collecting food and drink information so as not to encourage calorie counting in this population). The research assistant became “teammates” via the app with each member of the dyad to track progress and tailor follow-up phone call interviews based on tracker information collected during the interim between participant contact. At the orientation, the research assistant also reviewed local community resources for PA, the Office of the US President’s Council on Sports, Fitness & Nutrition (PCSFN) recommendations, and an electronic healthy recipe resource [21].

Daily step count and active minutes were reviewed within the Jawbone smartphone app and then manually recorded by the research assistant into a study tracker diary. These data, along with age-specific goals and personalized fitness goals, drove algorithmic motivational telephone scripts after weeks 1, 2, 4, and 8. At each of these sessions, the research assistant used these data to set daily personalized step-count and active-minutes goals with each participant within the dyad. Goals were established as follows. (1) If the participant reached the national guidelines for age (as defined by the Centers for Disease Control and Prevention [CDC] and the PCSFN for active minutes, and by PCSFN for daily step count), they were encouraged to continue to perform at this level. (2) If they met their previous goal but still were not reaching the national guidelines for age, they were recommended to walk an average of 1000 additional daily steps for the following week or to add an average of 5 active minutes per day for the following week, or both. (3) If they did not meet their previous goal, they were recommended to try to add 1000 daily steps or to add an average of 5 active minutes per day for the following week, or both. Additionally, the research assistant and participants worked together to identify new activities and exercises to try both alone and with their parent. Each of these sessions (after weeks 1, 2, 4, and 8) included a follow-up survey with reflective questions to address noncompliance issues in terms of PA and tracker use and plans for PA during the intervening time between study contacts. We also sent a brief email reminder at week 6 with a review of each participant’s fitness goals and the study contact information in case the participant experienced any issues or concerns. In a final debriefing, either via phone or in person, both parents and adolescents completed another set of questions about their current exercise and PA behaviors, barriers to change, perceptions about exercise and health, and fitness goals. As an incentive to participants, we gave each parent and adolescent a US $20 gift card and let them keep their activity trackers in exchange for their successful study participation. We conducted a follow-up interview with participating parents a few months later to determine their sustainment of PA changes.

**Table 1:** Jawbone UP MOVE app activity tracker setup.

Setting	Description
Goal: steps	8500 steps (parent) 12,000 steps (adolescent) This is the recommended goal based on the Office of the President’s Council on Sports, Fitness & Nutrition.
Goal: weight	Current weight This was not a weight loss intervention; this was an exercise intervention. We did not want the participants to become overly focused on or discouraged by weight goals that are not realistic to accomplish in a 10-week study.
Privacy and sharing	Sleep, steps, and workouts We were not collecting the following information through sharing: food and drink information, weight changes, and mood. This was primarily a physical activity–based intervention. We did not want adolescents focused on their weight and counting calories as the primary aim of this study. Therefore, we were not collecting or encouraging the dyads to record that information. We offered a healthy recipe website as a resource as a part of the intervention independent of the Jawbone app.
Notifications	Smart coach, team activity, duels, move goal, workout summary, daily sleep recap, and battery level. We used all the notifications because we knew that some participants may want all these features and later change them to access them. We did not want some participants to have access to certain features and others not.
User settings	Standard settings: reported height, reported weight, sex, date of birth.

### Measures

Primary outcomes were the percentage of successful days, defined as days when participants reached either their personalized daily goals or those set for their age by the national guidelines (average daily step counts defined by the PCSFN and daily active-minutes goals defined by both the CDC and the PCSFN). We defined this as either their personalized goal set with the research assistant during their last motivational interview or their ability to achieve the national guidelines goal of 8500 steps and 30 minutes for parents or 12,000 steps and 60 minutes for adolescents. A secondary analysis explored using a reduced step-count goal for adolescent participants to 10,000 steps per day or their personalized set goal if their average step count was less than 10,000 steps per day. This secondary analysis was based on research that evaluated step-count goals specifically for overweight adolescents [3,11]. We also calculated average daily steps and average daily active minutes for both parents and adolescents. This measure was calculated using captured data from the activity tracker smartphone app and dividing the total number of steps or active minutes by the number of days the activity tracker was worn. That measure showed incredible variability and was affected by compliance issues, particularly low daily step counts that were counted but likely reflected poor compliance on a given day.

Additional secondary outcomes were activity tracker compliance, self-reported weight change, parental and adolescent perceptions of overall health and fitness level, barriers to PA, evaluation of program efficacy, usefulness and appeal, and effect of the intervention on bonding within the parent-adolescent dyad. Such measures were not previously validated but are original questions that we developed based on key informant input from a group comprising a psychologist, 2 pediatricians, and an epidemiologist. We adopted self-reported preintervention PA questions from the Women’s Health Initiative Physical Activity Questionnaire [22]. We entered data into the StudyTRAX research platform (Studytrax, Macon, GA, USA) manually from paper copies of surveys and from the research assistant’s review of daily activity tracker data. Double data entry was performed for all data by 2 independent, trained research assistants. Any discrepancies were addressed by reviewing the paper copies or the smartphone app data.

### Statistical Analysis

We calculated descriptive statistics for characteristics and outcome measures: frequencies, percentages for categorical variables, and mean (SD) for continuous variables. Due to the small sample size and the exploratory nature of this pilot study, we conducted all statistical analyses using nonparametric methods; for example we also evaluated dyad correlations using Spearman rank order correlations. Dyad correlations used weekly averages. We adjusted *P* values using the Bonferroni method within the same family of hypotheses and considered *P* values less than .05 to be statistically significant. All analyses were performed using SAS v9.4 (SAS Institute).

## Results

### Participant Demographics

Table 2 presents study participant demographics. A total of 9 adolescents (4 female and 5 male) and parent dyads (all female) participated. Interestingly, all parent participants in the dyads were mothers. The adolescents’ mean age was 15 (SD 0.9) years (range 14-16); the parents’ mean age was 47 (SD 8.0) years (range 36-66). One adolescent and 1 parent were of Hispanic or Latino origin. In terms of race, 5 adolescents categorized themselves as white, 2 as black or African American, and 2 as other, with corresponding results for the parents. Self-reported mean weight loss over the 10-week intervention period for adolescents was 4.3 (SD 5.1) lb (2.0 [SD 2.3] kg); adjusted *P*=.56, and self-reported mean weight loss for parents was 9.1 (SD 9.2) lb (4.1 [SD 4.2] kg); adjusted *P*=.08. All parents and adolescents described their perceived weight at the end of the intervention as either the same as or improved over the start of the intervention. Self-reported baseline PA data from a preintervention survey showed that only 22% (2/9) of adolescents and no parents endorsed doing moderate to vigorous activity for 5 days per week or more at the start of the study. Of the 9 parents, 7 reported doing moderate to vigorous activity on zero days of the week. Only 33% (3/9) of adolescents and 11% (1/9) of parents reported that they did an hour or more of moderate to vigorous exercise when they did engage in it at all.

**Table 2:** Demographic characteristics of adolescents and their parents participating in the study.

Characteristics		Adolescents, n (%)	Parents, n (%)
**Sex**			
	Male	4 (44)	0 (0)
	Female	5 (56)	9 (100)
**Race**			
	Black or African American	2 (22)	2 (22)
	White	5 (56)	5 (52)
	Other	2 (22)	2 (22)
**Ethnicity**			
	Hispanic or Latino	1 (11)	1 (11)
	Non-Hispanic or Latino	8 (89)	8 (89)

**Table 3:** Weekly success rate of step count and active minutes for adolescents^a^.

Week	Percent step success, mean (SD)			Percent active-minutes success, mean (SD)	
	12,000 steps or personalized goal	10,000 steps or personalized goal	Personalized goal only	60 minutes or personalized goal	Personalized goal only
1	18 (20)	32 (34)	17 (38)	70 (26)	71 (46)
2	39 (27)	41 (29)	40 (40)	52 (32)	49 (50)
3	36 (28)	39 (27)	37 (49)	59 (23)	57 (50)
4	49 (40)	49 (40)	50 (50)	61 (29)	61 (49)
5	42 (24)	46 (22)	41 (50)	48 (23)	50 (51)
6	31 (39)	31 (39)	32 (47)	49 (31)	55 (50)
7	24 (35)	24 (35)	25 (44)	51 (31)	48 (50)
8	19 (28)	26 (36)	22 (42)	34 (29)	39 (49)
9	35 (40)	35 (40)	44 (50)	44 (28)	53 (51)
10	46 (41)	46 (41)	52 (51)	50 (41)	60 (50)
Total	35 (19)	39 (20)	35 (48)	55 (17)	55 (50)

^a^Raw data are available on request from the corresponding author.

**Table 4:** Weekly success rate of step count and active minutes for parents^a^.

Week	Percent step success, mean (SD)		Percent active-minutes success, mean (SD)	
	8500 steps or personalized goal	Personalized goal only	30 minutes or personalized goal	Personalized goal only
1	21 (25)	21 (41)	79 (32)	82 (39)
2	47 (28)	46 (50)	80 (20)	82 (39)
3	26 (28)	26 (44)	81 (16)	84 (37)
4	31 (35)	31 (46)	84 (15)	84 (37)
5	53 (32)	53 (50)	89 (16)	90 (30)
6	51 (35)	53 (50)	91 (13)	93 (26)
7	51 (35)	52 (50)	87 (18)	89 (32)
8	45 (39)	42 (50)	88 (25)	89 (32)
9	41 (25)	40 (49)	68 (31)	67 (48)
10	35 (41)	36 (48)	63 (39)	67 (48)
Total	39 (24)	40 (49)	81 (16)	83 (38)

^a^Raw data are available on request from the corresponding author.

### Success in Meeting Goals

Table 3 and Table 4 list the number of successful days for both average daily step count and average daily active minutes for the participants. On average, adolescents met their personalized daily step-count goal or the minimum goal set by the PCSFN on 35% (range 11%-62%) of the days they wore the activity tracker. Parents met the personalized daily step-count goal or the PCSFN goal on 39% (range 3%-68%) of the days that they wore their activity tracker; on 55% of the days, adolescents (range 27%-85%), and on 83% of days, parents (range 52%-97%) met their active-minutes goals. A secondary analysis of the adolescents’ step count using either the 10,000 step or the personalized step-count goal showed that adolescents met their personalized step-count goal on 39% (12%-72%) of days they wore a tracker. All participants reached more recent literature-supported step counts (10,000 for all children, 8500 for adults) at some point in the intervention but were unable to sustain these step counts consistently.

**Figure 1:**
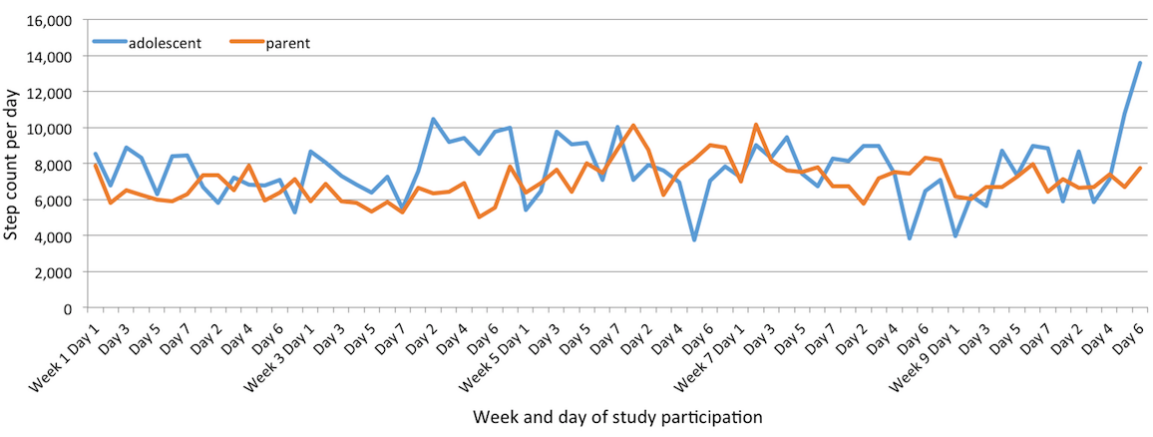
Mean daily step counts for adolescents and parents.

**Figure 2:**
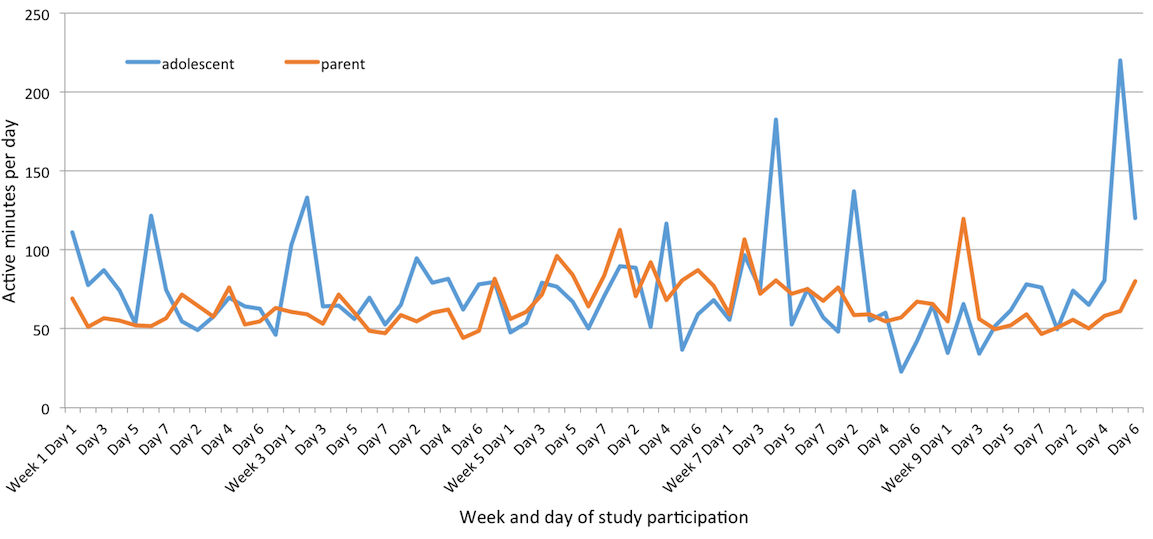
Mean daily active minutes for adolescents and parents.

Figure 1 shows mean daily step counts and Figure 2 shows mean daily active minutes for all participants. By the last week, adolescents had an average daily active minutes total of 101 and parents had an average of 59 minutes over the course of that week, both well above the national guidelines (CDC and PCSFN) for their respective age groups. For average daily step count over the course of the final week, the adolescent group averaged 8652 steps per day and parents averaged 7044 steps per day.

**Figure 3:**
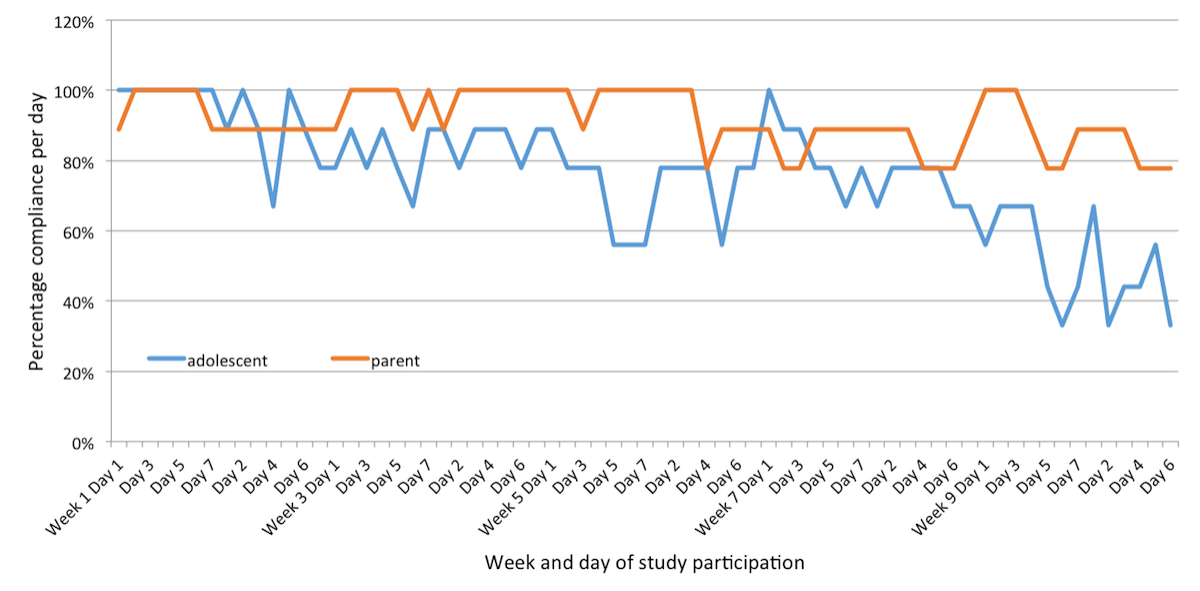
Percentage daily compliance for adolescents and parents.

Most importantly, weekly parent and adolescent step count and active minutes were significantly correlated (step count: *r*=.36, adjusted *P*=.002; active minutes: *r*=.30, *P*=.007); children of parents who were more successful in achieving their goals were also more successful in achieving their own goals. While overall parent and adolescent PA was significantly correlated, there was much day-to-day variability, as Figure 1 shows. Parental age was inversely correlated with step-count success (*r*=–.78, *P*=.01). While activity tracker compliance was the primary study barrier, it was fairly high for a behavioral intervention, particularly for the parents. On average, parents wore their tracker 92% of the time and adolescents wore their trackers 76% of the time (Figure 3). The 3 adolescents with the lowest step-count performance noted barriers to exercise (2 noted time, 1 noted feeling tired), while all other adolescents noted no barriers to exercise.

### Postintervention Survey

In the postintervention survey, which was completed by 8 of the 9 parents who participated in the program, walking was the most common exercise added to the participants’ exercise regimen since starting the program. Of the 8 adolescents who completed a postintervention survey, 7 stated that they added walking to their PA regimen. A total of 6 of 8 parents stated that they had started walking with their children regularly since starting the program. Cycling was the second most common new exercise added by parents and adolescents, with swimming, running, and aerobics as other mentioned activities. Of note, neither cycling nor swimming could be recorded as an activity by the study-selected tracker.

In terms of overall program satisfaction, both parents and adolescents enjoyed the program, rating multiple areas of content helpful on a postintervention survey. Both adolescents and parents rated the program as being most helpful in the following ways: improving health, having fun, and spending time with the other member of the parent-adolescent dyad. When rating programmatic components on a scale of usefulness from 1 to 5, participants rated items that were tailored or personalized (ie, in-person orientation, motivational phone calls) more highly than generic items (ie, healthy recipe websites, community fitness handouts). Parents were more likely to feel comfortable keeping up with the changes they had made, rating themselves an average of 8 out of 10 on a likelihood scale. Adolescents were less confident in maintaining the changes, rating their likelihood to maintain change as 5 out of 10.

These results were underscored again in a brief follow-up questionnaire completed by 7 parents approximately 3 months after the intervention ended. All parents (7/7) reported that they had made changes to their overall health and fitness routine as a result of the study. Of these 7 parents who completed the follow-up interview, 6 stated that their children had made changes to their overall health and fitness routine as a result of the study, and 5 parents noted that other family members had made changes as a result of the parent-adolescent dyad participating in the study. Changes mentioned included increased health awareness, increased walking or joining a gym, and healthier diets. A total of 5 of the adolescents used the tracker for some period of time after the study (ranging from a few weeks through continued use at the time of questionnaire completion). The most common reasons for discontinuation were lost trackers, the desire to purchase a different brand of activity tracker, and broken bands.

## Discussion

### Principal Findings

Our study found that both adolescents and parents achieved step goals at least a third of the time (35% and 39%, respectively) and active-minutes goals more than half of the time (55% and 83%, respectively), with both percentages for step count and active minutes being higher in the parent group. Most importantly, we found that parents’ and adolescents’ step-count and active-minutes success rates were significantly correlated.

A review of daily step count and active minutes showed great variability. Both adolescents and parents met US national guidelines’ daily active-minutes goals across week 10 but were not at the national guidelines for daily step count. The variability and lower step-count performance were likely affected by compliance issues and the inability of the activity tracker to capture all activities in the form of a step count.

### Comparison With Prior Work

Prior studies have shown that, in general, PA decreases during adolescence [4-6]. In 1 study, adherence to PA guidelines for active minutes fell from 49% (6- to 11-year-olds) to 12% (12- to 15-year-olds) in boys and from 35% (6- to 11-year-olds) to 3% (12- to 15-year-olds) for girls [5]. Another study of PA by Nader et al found that, by 15 years of age, adolescents were engaging in moderate to vigorous PA for only 49 minutes per weekday and 35 minutes per weekend day [6]. Beets and colleagues found in a study of PA among children in 13 countries that mean steps per day decreased through adolescence to a value of approximately 8000 to 9000 steps per day by age 18 years [23]. A US National Health and Nutrition Examination Survey review of the 2005-2006 data cycle found a similar decline in adolescence with average daily step counts at age 16 years of 9376 for girls and 10,668 for boys [24]. These studies indicate that reaching the formal national guidelines for PA is an incredible challenge in the average adolescent population. Therefore, the fact that our participants, who were in a higher risk category, reached their set goal, or that of the national guidelines, at least a third of the time for step count and greater than half of the time for active minutes is promising. The discrepancy in the 2 PA measures (step count vs active minutes) likely reflects the inability of the tracker to capture all PA activities, such as swimming and biking, in terms of step count. Dyads were able to manually enter the time that they participated in these activities toward their active minutes for the day.

While national guidelines formally recommend 60 minutes of daily PA and 12,000 steps for children [7-10], Janssen and Leblanc’s systematic review found that just a few hours a week of PA could be effective, particularly for high-risk groups such as ours [3]. Additionally, multiple studies have suggested that 10,000 to 11,700 steps might be a more appropriate range for adolescents, especially for overweight adolescents such as those participating in this pilot study [11,25]. In light of this additional research, we revisited the adolescents’ success rates using a goal of 10,000 steps per day. In these supplemental analyses, by decreasing the step-count target to this revised level, we were able to demonstrate that the performance of our participants was slightly higher, with 38% successful days in the 10,000 step-count group.

The most interesting finding of our study is the strong correlation of both step-count and active-minutes success for the parent and adolescent as a dyad. The existing literature on parent and child or adolescent PA correlations is limited, has mixed findings, and often has focused on younger populations [17,26]. McMurray and colleagues found that, in 7- to 10-year-old children, parent and child PA levels were correlated, especially on weekend days [17]. Jago and colleagues found that, while sedentary time of parents and children is correlated, PA was not [26]. Both of these studies focused on younger populations. Our study is unique in that it focused on adolescents, who are at a crucial tipping point in their PA habits. Additionally, our study paired parents and adolescents together to create a framework of mutual support, which is also supported by the literature [27,28]. Pyper et al found that 3 parent support behaviors contributed to predict their child’s PA: taking their child to places to be more active, encouraging their child to be active outdoors, and taking part in PA with their child. Of these 3 behaviors, taking part in PA with their child was the least-reported behavior in their study and the one the authors suggested needs to be explored most [28]. While it is likely that all 3 of these behaviors occurred in the context of our study, the third behavior is most central to the framework of our intervention.

Our study is also unique in that it is, to our knowledge, one of only a few studies to date that evaluated the use of wearable activity trackers in the adolescent population [29,30]. One such study that is underway in Australia, and whose results are not yet available, is the Raising Awareness of Physical Activity (RAW-PA) study. Like our study, the RAW-PA will use wearable activity trackers in the adolescent population to look at the impact on PA [31]. However, our study is unique in that we looked at the role of these devices for both the adolescent and their parent in an intervention framed around parent-adolescent dyads.

### Limitations

Limitations of our study include the small sample size, lack of substantial baseline PA data, and issues related to compliance with the wearable activity trackers. While compliance was a primary barrier to data capture, overall tracker compliance was similar to that in other studies of adolescent activity tracker use [30,32]. Common problems included issues with syncing the tracker to the Jawbone UP MOVE app to record data, lost trackers (n=5) and broken tracker bands (every dyad had a physically broken band at some point, with approximately 15 broken during the study). These common issues led to missing data for multiple participants. The activity trackers were not sophisticated enough to be able to record certain activities. Our population reported both bicycle riding and swimming as common activities, and these often were not accurately reflected in step count, which could potentially result in underreporting of PA. Since the trackers were not waterproof, participants reported having to remove the tracker for any water sports, such as swimming or playing at the beach, which also potentially resulted in underreporting of activity, since participation spanned the summer months. Dyads were instructed to record these activities as active-minutes time, but not all did so consistently. We hypothesize that, if more of these activities had been captured, the success rate would have been much higher, particularly for step-count percentages. Additionally, it was difficult to assess whether low step count reflected low activity or partial compliance, which may have contributed to underreporting as well.

Baseline PA data are difficult to obtain in any activity tracker study without complicating the results. A study in older adults by Gualtieri et al showed that activity trackers as a concrete reminder can alone increase motivation for PA in research participants [33]. Additionally, as this was a small pilot study and not a randomized controlled trial, we used self-reported measures of baseline PA data. However, having some quantitative baseline PA data will be important for future studies and may require a “burn-in” period where participants are advised to wear the activity tracker and follow their normal routine without the other additional motivation or mutual support mechanisms.

The lack of automation for recording tracker data and interview results created time-intensive processes requiring substantial personnel resources to conduct the tailored motivational interviews, ultimately limiting the number of participants that could be recruited at any one time and the number of encounters for motivational interviewing. These fewer interactions between participants and study personnel often translated into lost opportunities to address tracker compliance issues or to motivate participants to sustain their step-count and activity goals when they made them. Additionally, weight information was self-reported due to our inability to bring participants back at multiple intervals. Based on lessons learned, for future studies, we are looking to provide families with home-based smartscales that integrate with the wearable trackers to obtain accurate weight changes during the intervention period.

### Conclusions

Our findings indicate that parent-adolescent dyads have highly correlated PA success rates. This supports further investigation of family-centered weight management interventions for adolescents, particularly those that involve the parent and the adolescent working together. Future studies will include a more detailed account of baseline PA data prior to distribution of the activity trackers, followed by a 1-week period of asking participants to wear their tracker without any motivation or goal setting to obtain some objective quantitative information about their baseline activity. Additionally, the next steps will be to automate the compilation of tracker data and enhance the process of tailored motivation through the development of an app. This more intensive interaction will provide for more rapid intervention for adherence and goal setting and maintenance. While the step-count and active-minutes features from the Jawbone remain on most commercially available trackers, we would use more sophisticated activity trackers. Such trackers will be more durable and waterproof, thus enhancing compliance and complete collection of activity data across the spectrum of activities that adolescents and their parents engage in throughout the year.

## Abbreviations

CDC: Centers for Disease Control and Prevention
PA: physical activity
PCSFN: President’s Council on Sports, Fitness & Nutrition
RAW-PA: Raising Awareness of Physical Activity
